# Urban Blisters

**Published:** 2013-03-15

**Authors:** Nicholas Chang, Rebecca Nunn, Stephen M Milner, Leigh Ann Price

**Affiliations:** Department of Plastic and Reconstructive Surgery, Johns Hopkins University School of Medicine, Baltimore, MD

**Figure:**
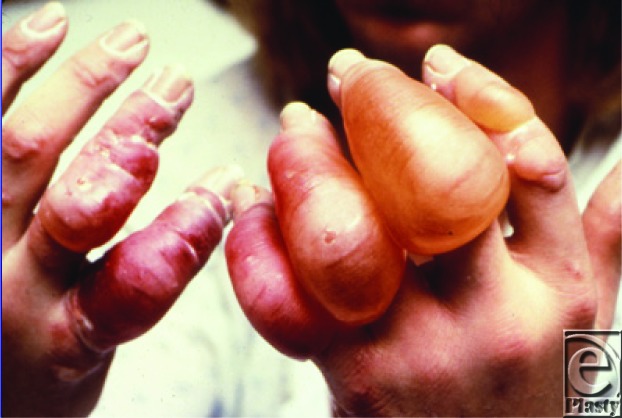


## DESCRIPTION

A 12-year-old girl presented to the emergency department after waiting at a bus stop in winter.

## QUESTIONS

1. **What is the diagnosis?**
2. **What is the mechanism of injury?**
3. **How would you treat this patient?**
4. **What are some of the long-term sequelae of this injury?**

## DISCUSSION

This girl has frostbite. Large, clear fluid-filled blisters are seen over the dorsum of the fingers.

Frostbite is a severe, cold-induced injury that causes localized damage to skin and underlying tissue. Tissue destruction can be attributed to an ischemia reperfusion injury. Following exposure to subfreezing temperatures, there is an immediate cell death due to extracellular ice crystal formation. As the tissue is rewarmed, ice crystals form in the intracellular and extracellular compartments furthering damage and ischemia. A massive release of chemical and inflammatory mediators (such as, thromboxane A2, prostaglandin F2α, histamine, and bradykinin) cause endothelial damage, vasoconstriction, and platelet aggregation. This increases capillary permeability and causes microthrombi, further potentiating ischemia.^1^

Treatment of frostbite can be subdivided into 3 phases: (1) prethaw phase, (2) immediate hospital (rewarming) phase, and (3) postthaw phase.^2^ The primary goal of the prethaw is to remove the patient from the cold environment, and to protect the affected area. If there is no risk of refreezing prior to reaching a hospital, rewarming can be attempted using warm water or body heat (eg, placing affected digits in the axillae). Rewarming from sources of dry heat (eg, stoves or fires) or rubbing the affected area should be avoided to prevent thermal injury and mechanical trauma, respectively.^3^ Do not attempt rewarming if there is a chance of refreezing as this will cause further damage to the tissue. Once in hospital, definitive care can be initiated to improve tissue viability and prevent further sequelae. Rewarming is achieved by placing the affected tissue in warm water (ideally in a warm bath or whirlpool at 40° C-42° C, but under 44° C). Thawing is complete when the skin turns red/purple and is soft to touch. This normally takes around 15 to 30 minutes.^2^ The postthaw phase includes standard burn wound care, analgesia, tetanus prophylaxis, and/or surgery (eg, tissue debridement, escharotomy, fasciotomy, delayed amputation).^4^ Debridement of the clear blisters is completed to remove the local tissue thromboxanes, but leaving the hemorrhagic ones intact as they are often deeper within the tissue and offer protection. The extremities should be elevated to decrease the swelling and the hands splinted in the intrinsic plus position. Topical application of Aloe vera (a thromboxane inhibitor) to the wound every 6 hours is often helpful to fight against the local vasoconstrictive effects of thromboxane. Oral ibuprofen (a cyclooxygenase inhibitor) is given to minimize the systemic effects of thromboxane and decrease the secondary tissue damage. There is some evidence that thrombolysis given in the acute stage may reduce the risk of surgical amputation in severe deep frostbite injuries.^1^

Complications may present early or late. Infection, gangrene, and autoamputation may present in the short-term. Numbness, loss of fine motor control, and a throbbing or tingling sensation may persist from days to weeks. Long-term sequelae include scarring, tissue atrophy, cold hypersensitivity, and peripheral neuropathy.^1^
